# Easily doped *p*-type, low hole effective mass, transparent oxides

**DOI:** 10.1038/srep20446

**Published:** 2016-02-08

**Authors:** Nasrin Sarmadian, Rolando Saniz, Bart Partoens, Dirk Lamoen

**Affiliations:** 1 CMT, Departement Fysica, Universiteit Antwerpen, Groenenborgerlaan 171, B-2020 Antwerpen, Belgium; 2 EMAT, Departement Fysica, Universiteit Antwerpen, Groenenborgerlaan 171, B-2020 Antwerpen, Belgium

## Abstract

Fulfillment of the promise of transparent electronics has been hindered until now largely by the lack of semiconductors that can be doped *p*-type in a stable way, and that at the same time present high hole mobility and are highly transparent in the visible spectrum. Here, a high-throughput study based on first-principles methods reveals four oxides, namely X_2_SeO_2_, with X = La, Pr, Nd, and Gd, which are unique in that they exhibit excellent characteristics for transparent electronic device applications – i.e., a direct band gap larger than 3.1 eV, an average hole effective mass below the electron rest mass, and good *p*-type dopability. Furthermore, for La_2_SeO_2_ it is explicitly shown that Na impurities substituting La are shallow acceptors in moderate to strong anion-rich growth conditions, with low formation energy, and that they will not be compensated by anion vacancies *V*_O_ or *V*_Se_.

Although *p*-type transparent conducting oxides (TCOs) have already made their way into optoelectronic devices such as light emitting diodes (LEDs), transparent thin film transistors, and solar cells^1^,^2^,^3^, current *p*-type TCOs are unable to match the performance of *n*-type TCOs in terms of charge carrier mobility and/or optical transparency^1^,^4^,^5^,^6^. This has significantly interfered with device efficiency or with the development of advanced applications based on active transparent electronic components, such as bipolar transistors or diodes. Thus, it is of great interest to find new *p*-type TCO compounds, with properties that would lead to a technological breakthrough and herald the real era of transparent electronics^7^. Still, in spite of considerable research efforts for more than a decade, this goal has not yet been reached^1^,^4^,^8^. The data on established *p*-type TCOs compiled in Table 2.1 of ref. 7 (page 10) are quite illustrative. Compounds exhibiting a good transparency (above 70%), tend to show very low conductivities, typically below 1 S cm^−1^, while those with a conductivity above 10 S cm^−1^ have a poor optical transparency (below 30%). In comparison, Sn-doped In_2_O_3_, the most widely used *n*-type TCO, has an optical transparency above 80% and a conductivity of the order of 10^4^ S cm^−1^ 9. The two intrinsic properties of a host TCO material that are critical in this regard are its band gap and the charge carrier effective mass. The former should be wide enough that visible light (frequencies ranging roughly from 1.59 to 3.18 eV^10^) is not absorbed and the latter should be conducive to a good charge carrier mobility. In the case of In_2_O_3_, the band gap is of 3.75 eV and the electron effective mass of ~0.35 *m*_*e*_ (with mobilities ranging from 20 to 50 cm^2^V^−1^s^−1^, depending on carrier density)^9^. However, it is important to realise that a wide band gap and a low hole effective mass do not readily promise a good *p*-type TCO. A clear example of this is given by ZnO (a very good *n*-type TCO when doped, e.g., with aluminum^11^). Indeed, the hole effective mass values for ZnO range from 0.31 *m*_*e*_ to 0.59 *m*_*e*_, depending on direction and band^12^. For this reason, and because of its good transparency, there have been continuous attempts to obtain *p*-type doped ZnO. These efforts have resulted in *p*-type conductivity in doped ZnO being announced several times in the past, even with mobilities rivaling those of *n*-type TCOs^11^. Unfortunately, it was invariably found later on that the *p*-type conductivity was unstable, i.e., the acceptors were eventually compensated, typically by donor native defects^11^.

The above underlines the notorious difficulty in finding a transparent oxide that has a low hole effective mass and that at the same time can be doped *p*-type in a stable way. The reasons for this are fairly understood and have been discussed by previous authors^4^,^13^. Briefly, the states around the valence band maximum (VBM) in oxides are typically of oxygen 2*p* character, which has two implications. First, these states tend to be localised, so the dispersion around the VBM is low and, consequently, the hole effective mas is large. Second, the VBM tends to be deep below the vacuum level, i.e., the ionisation potential is large. Thus, finding shallow acceptors is difficult and/or these will tend to be compensated. To solve this conundrum one may consider alloying with an element with 3*d* orbitals close to the oxygen 2*p* orbitals, so that hybridisation lifts the VBM, making *p*-type doping feasible and lowering the effective hole band mass. This was the original idea behind the first *p*-type TCO to be produced, CuAlO_2_^4^. Another idea, again put forward by Hosono and co-workers^4^, is to replace oxygen with a chalcogen (S, Se, or Te), which have more delocalised *p* orbitals. This led to the discovery of LaCuOS and LaCuOSe as *p*-type oxides^4^. However, in the former mobility is poor, while in the latter transparency is insufficient because of its smaller gap^14^,^15^. Recently, thanks to their high-throughput work, Hautier and co-workers^16^ found that the presence of pnictogens, such as P and As, can result in a low hole mass as well. They also found that this can occur if the oxygen 2*p* orbitals can hybridise with *s* orbitals, in addition to closed shell *d* orbitals [i.e., (*n* − 1)*d*^10^*ns*^2^ orbitals]. This is the case, for instance, of A_2_Sn_2_O_3_ (A = K, Na), K_2_Pb_2_O_3_, and PbTiO_3_. However, a theoretical study based on the *GW* approximation^17^,^18^ (calculation of the eigenvalues based on many body perturbation theory) indicates that, with a band gap ≤2.6 eV, the first three present an insufficient transparency^16^. On the other hand, following the same approximation, the band gap of PbTiO_3_ was found to be 3.7 eV, but its stable *p*-type dopability is uncertain^16^.

In light of all the research efforts mentioned, the question is how to proceed to try to identify, as efficiently as possible, materials with a wide enough band gap, a low hole effective mass, and which can be easily doped *p*-type, i.e., in which *p*-type conductivity will be stable. Indeed, given the very large number of existing oxides that can be studied, or possible chemical modifications that can be made, a systematic experimental study is not possible. High-throughput *ab initio* computations, on the other hand, can be used to screen large classes of materials, searching for those that exhibit a predetermined basic set of properties, qualifying them as potential candidates for a specific application^19^,^20^. This approach has already been used in the search for novel organic *p*-type semiconductors (not transparent)^21^ and candidate TCO materials^16^,^22^, as well as new thermoelectric^23^, piezoelectric^24^ and scintillator materials^25^.

In this work we use a high-throughput search engine to screen all the binary, ternary, and quaternary oxides reported in the AFLOWLIB computational database^26^,^27^ (12211 oxides in all), and make a first selection of compounds with a wide band gap and low hole effective mass. Systematic higher-accuracy first-principles electronic structure calculations allow us subsequently to obtain a final list of four oxides that we predict to be easily doped *p*-type, while being transparent nominally in the entire visible range and having an effective hole mass lower than 1 *m*_*e*_, namely La_2_SeO_2_, Pr_2_SeO_2_, Nd_2_SeO_2_, Gd_2_SeO_2_. For demonstration, we consider La_2_SeO_2_ and show that Na impurities substituting La (Na_L*a*_) in this material are shallow acceptors in moderate to strong anion-rich conditions, with a low formation energy, and that they will not be compensated by anion vacancies *V*_O_ or *V*_S*e*_.

## Results

### Database screening

The AFLOWLIB is an extensive repository of computational data on materials, including structural and electronic properties. We wrote a Python-based search engine to screen the hole effective masses and band gaps of all the binary (1885), ternary (6416), and quaternary (3910) oxides in the AFLOWLIB database, all of them completely identified compounds from the Inorganic Crystal Structure Database (ICSD)^28^. To increase the chances that our final compounds will present a comparably good hole mobility, we select those oxides with an average effective hole mass ≤1 *m*_*e*_, which is considerably lower than in most of the current *p*-type TCOS^6^,^29^. The effective masses reported in the AFLOWLIB database are averages computed taking into account all symmetry considerations and contributions from bands falling within 26 meV of the band edges^25^,^26^, providing thus a reliable estimate. With respect to the band gap, we note that the value reported in the AFLOWLIB database is semiempirical, resulting from a combination first-principles computations and a least-squares fit to the experimental gaps of a set of 100 selected compounds. Compared to experiment, the reported fitted values present a percentage error root-mean-square of 24%^25^, which is quite reasonable for such a large high-throughput database. However, because of the margin of error, and to try to avoid discarding oxides that experimentally might in fact be transparent in the whole visible range, we set a lower limit of 2.5 eV for the fitted band gap to accept an oxide in our selection at this stage.

**Figure 1:**
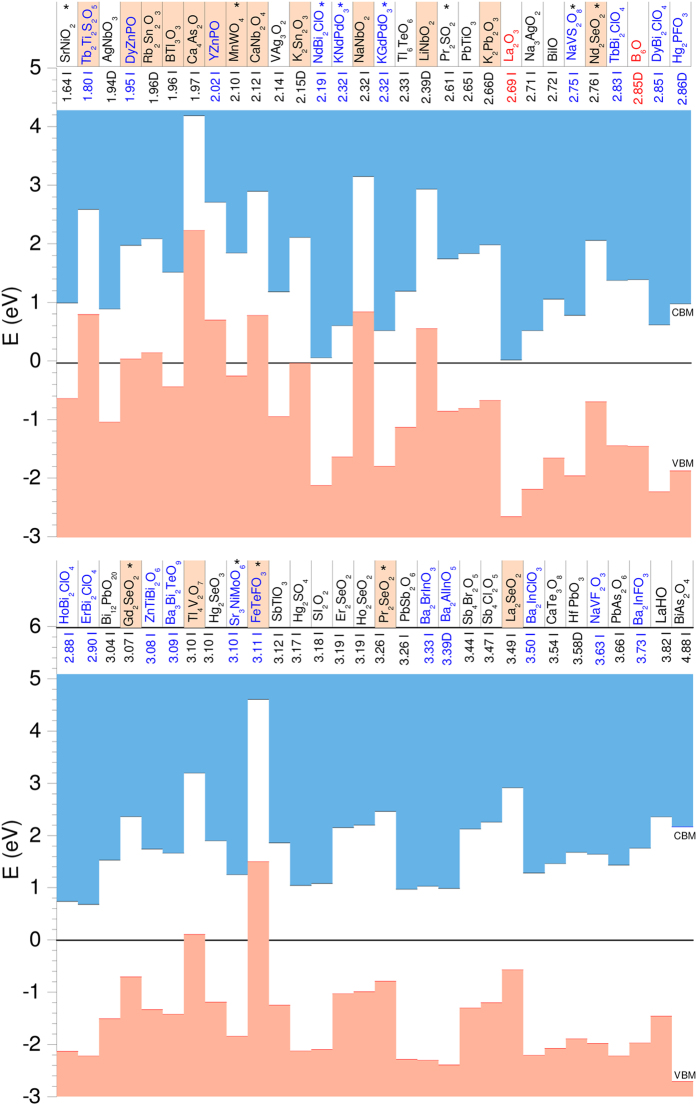
Valence and conduction band energies with respect to the branch point energy. The oxides are arranged in ascending order according to their HSE06 band gap value, a “D” (“I”) indicating a direct (indirect) fundamental gap. The highlighted oxides are those that are easily doped *p*-type, according to our criterion (see text). Quaternary oxides are written in blue, ternaries in black, and binaries in red.

The above criteria result in a list of 2 binary oxides, 41 ternaries, and 27 quaternaries for which to proceed with higher accuracy electronic structure calculations in the next stage. However, we found that among these there are numerous oxides presenting magnetic order (ferromagnetic or antiferromagnetic). These entail calculations that are more involved and heavier than in the case of non-magnetic oxides. To reduce the number of such calculations, we screen out those oxides with magnetic order that at the same time contain elements that might raise toxicity concerns (i.e., As, Cd, Hg, Tl, Pb, and F). FeTeFO_3_ is screened out because its synthesis procedure likely involves toxic fluorine gas. Indeed, these are probably of less interest from an industrial perspective. Consequently, the list of oxides considered for further calculations consists of 2 binaries, 37 ternaries, and 21 quaternaries, i.e., 60 oxides in all. These oxides are catalogued in Fig. 1, with information discussed in the next Subsection. (See Supplementary Table S1 for the complete list of oxides, i.e., including those with potentially toxic elements, where we report the structural parameters for each compound in addition to their band gap values and effective masses.)

### *p*-type dopability screening

Determining the *p*-type (or *n*-type) dopability of a semiconductor using first-principles methods requires that the physics of defects is reliably accounted for and, accordingly, the electronic structure of the semiconductor, including the band gap value. Indeed, the *p*-type dopability of a semiconductor depends on whether acceptor-like defects are easily formed and whether it is prone to the spontaneous generation of compensating native defects (e.g., anion vacancies). Thus, the well known problem of the band gap underestimation by exchange-correlation functionals typically used in first-principles studies, such as the local density approximation (LDA) or the generalized gradient approximation (GGA), has been a hurdle until recently. Thanks to notable methodological developments in recent years, however, first-principles methods are capable today of a broader and deeper description of the physics of defects^30^, albeit at a greater computational cost. In the case of studies based on the Heyd, Scuseria, Ernzerhof (HSE06) hybrid-functional^31^,^32^, for instance, computational cost increases typically by an order of magnitude, or more, compared to the approximations just mentioned. Nevertheless, because of its reliability, the HSE06 hybrid-functional has practically become the functional of choice in the study of defects^30^, and is the one we use here (see the Methods section for further details).

A criterion with significant predictive power, indicating whether a wide band gap material is easily *n*- or *p*-dopable, is provided by the branch point energy (BPE) or charge neutrality level^33^,^34^,^35^. This is the energy level below which defect states in the gap will have a mainly donor-like, and above which they will have a mainly acceptor-like character. This is to say that it is the energy below (above) which the formation of donor (acceptor) defects becomes favorable. Hence, in a material with a BPE lying in the conduction band, or high in the band gap, donor impurity defects will tend to be shallow, while acceptor impurities will tend to be deep. Furthermore, it is also more likely that there will be native donor defects with very low formation energies for Fermi levels close to the VBM. As a consequence, even if a shallow acceptor impurity (or impurity complex) can be found, it will be difficult for it to have a sufficiently low formation energy as to avoid compensation by such native donors, which will act as killer defects (this is exactly the problem with ZnO, for which the BPE lies in the conduction band). Thus, to favor *p*-type doping, the BPE should lie instead below the midgap, and the lower the BPE, the easier *p*-type doping will be. To determine the BPE value we follow here the approach of Schleife and co-workers^36^,^37^, in which the BPE is calculated as the weighted average of the midgap energies over the Brillouin zone (cf. Methods for more details). The BPE depends solely on the bulk band structure, which makes it a computationally efficient screening tool.

In Fig. 1 we present the valence and conduction band energies of the oxides selected in the previous Subsection with respect to the BPE, for ease of comparison. The HSE06 calculated band gap values are given (a “D” or “I” indicating a direct or indirect fundamental gap), and the oxides are sorted in ascending order according to their gap value. Quaternaries are indicated in blue, while binaries are indicated in red. The position of the BPE with respect to the band edges allows us to classify the studied oxides in three classes. In the first class we have the oxides that in this work we consider to be easily *p*-type dopable, namely those for which the BPE lies in the valence band or in the lower fourth of the gap above the VBM. Oxides with a BPE above this limit might still be *p*-type dopable, but with our choice we try to ensure an easy *p*-type dopability. The compounds in this first class are highlighted in Fig. 1. There are fourteen ternary and four quaternary oxides in this class.

In the second class we classify the oxides for which the BPE lies above the CBM, or in the upper fourth of the gap below the CBM. These oxides are easily doped *n*-type. We do not focus on *n*-type TCOs in this work, and we just mention that La_2_O_3_, Na_3_AgO_2_ and the quaternaries KGdPdO_3_, and Bi_2_ClXO_4_, where X = Dy, Ho, Nd, or Er, belong to this class. Note that well known *n*-type TCOs, such as In_2_O_3_ or ZnO, did not make it to our list of oxides in Fig. 1 because they present a hole mass larger than 1 *m*_*e*_.

The third class consists of the rest of the oxides. Oxides for which the BPE is closer to the center of the band gap than to the band edges do not follow any obvious trend. Some may still be *n*- or *p*-type dopable, and some may be both (ambipolar). However, for oxides with a band gap, such as those considered here, this is probably difficult. Indeed, ambipolar doping becomes increasingly challenging the wider the band gap of a material because the electron affinity is becoming small and/or the ionisation energy is becoming large^13^,^34^,^38^. (Conversely, ambipolar doping is easy in narrower gap semiconductors. Si and Ge are among the best known examples^39^).

### *p*-type dopable, low hole mass, transparent oxides

As mentioned above, the AFLOWLIB band gap values present a margin of error, and it is important to verify the magnitude of the band gaps using a more accurate method. We find that while none of the *p*-type dopable oxides has an underestimated band gap in the AFLOWLIB database (this is the case for a few of the oxides in the other classes, such as BiAsO_4_ or ZnTiBi_2_O_6_; cf. Supplementary Table S1), there are several for which the gap is overestimated. Thus, for instance, LiNbO_2_ and K_2_Pb_2_O_3_ have direct band gaps that in the AFLOWLIB database are reported to be of 3.16 eV and 3.20 eV, respectively, but the corresponding HSE06 values are of 2.39 eV and 2.66 eV, respectively. Hence, although these oxides are *p*-type dopable and have a low hole effective mass, their complete transparency is not ensured. (Note that our finding regarding K_2_Pb_2_O_3_ agrees quite well with ref. 16.) On the other hand, one must take care of not discarding too rapidly an oxide with a low indirect fundamental gap, because it is direct transitions that are most important for transparency. Therefore, we screen our *p*-type dopable oxides searching for those with a HSE06 direct band gap band gap >3.1 eV. This is sufficient to ensure their nominal transparency in all the visible range. Indeed, if the direct band gap is symmetry-forbidden, the direct-allowed band gap will *a fortiori* be larger. Discarding oxides containing elements that might pose toxicity problems, this results in the list of four oxides in Table 1, X_2_SeO_2_, with X = La, Pr, Nd, and Gd. These oxides present excellent characteristics for *p*-type TCO applications, and are completely novel as such. For completeness, Table 1 includes the HSE06 values for the fundamental gap, the direct gap, the so-called second gap (we come back to this point in the Discussion section), the average hole mass reported in the AFLOWLIB, and the enthalpy of formation. We note that the last three oxides possess ferromagnetic order (for details, see ref. 40), which offers the opportunity to explore further applications of *p*-type TCO materials.

### *p*-type La_2_SeO_2_

To corroborate the assertion that the oxides in our final list are *p*-type dopable, we consider the case of La_2_SeO_2_ and show explicitly that Na will act as a shallow acceptor with charge state 2– and that anion vacancies will not act as hole killers in suitable growth conditions. The formation energy of a defect *D*, in charge state *q*, in a bulk compound is given by^41^


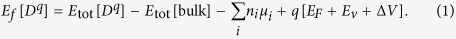


In the above, *E*_t*ot*_[*D*^*q*^] is the total energy of defect-containing system and *E*_t*ot*_[bulk] is the total energy of the defect-free system, *n*_*i*_ is the number of atoms of type *i* added to or removed from the system (*n*_*i*_ < 0 if the atom is removed and *n*_*i*_ > 0 if the atom is added), with *μ*_*i*_ the corresponding chemical potential. *E*_*F*_ is the electronic chemical potential, measured with respect to the VBM, *E*_*v*_, of the undoped system. Δ*V* is a reference alignment term (see the Methods section for further details).

**Figure 2:**
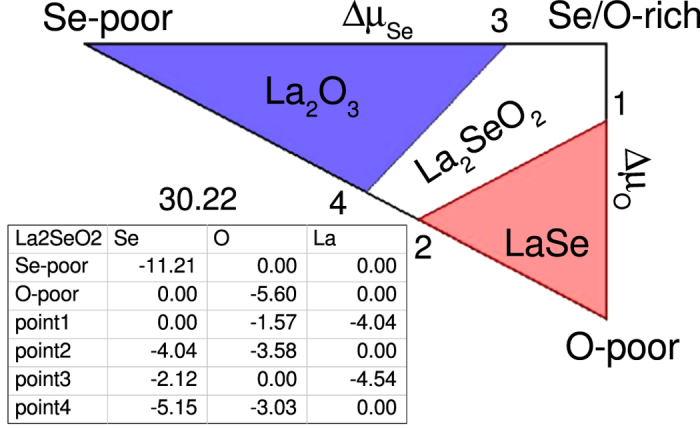
La_2_SeO_2_ stability triangle in the chemical potentials plane. The white area defines the range of chemical potentials in which La_2_SeO_2_ is stable against precipitation of competing binary phases La_2_O_3_ and LaSe. The chemical potentials are given with respect to their standard phases, i.e., 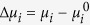, where 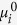 corresponds to the solid metal for selenium and to the diatomic molecule for oxygen.

**Figure 3:**
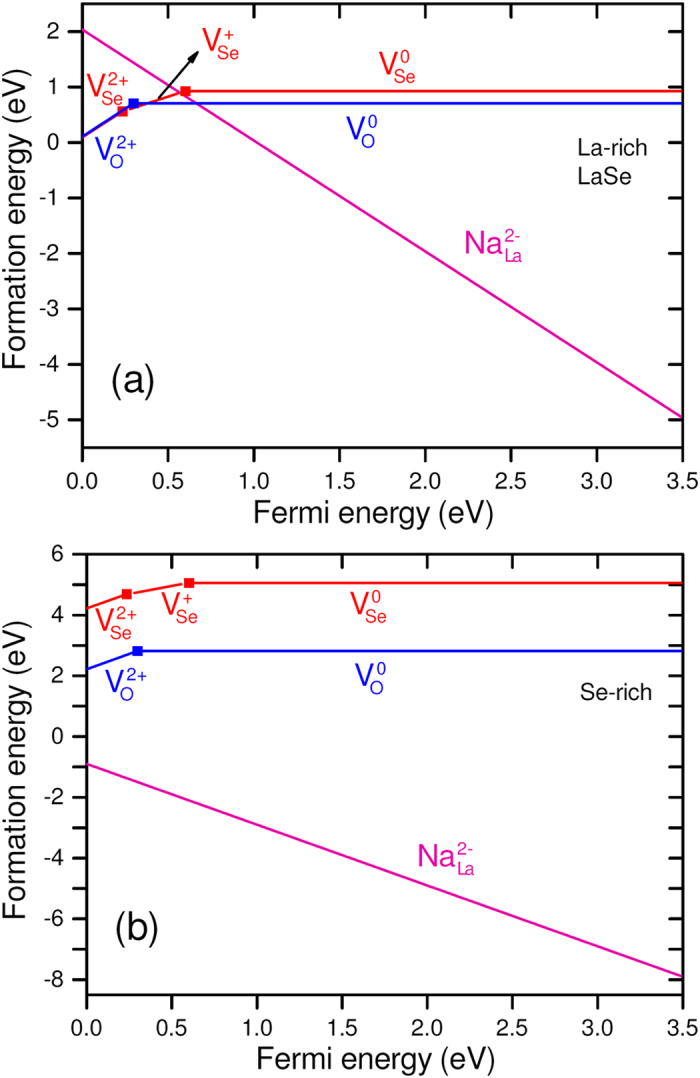
Formation energy of Na_La_, *V*_Se_ and *V*_O_ as a function of Fermi energy. For all defects only the charge state with lowest formation energy is shown at each Fermi energy value. (**a**) In La-rich conditions, 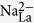 is a shallow acceptor, but will tend to be compensated by anion vacancies (*V*_S*e*_ and *V*_O_). (**b**) In Se-rich conditions 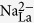 will not be compensated by anions vacancies and effectively dope La_2_SeO_2_ *p*-type.

The formation energy of a defect depends quite importantly on the chemical potentials of the atomic species involved. The relevant range of *μ*_*i*_ values is delimited by the stability of La_2_SeO_2_ against the precipitation of competing binary compounds La_2_O_3_ and LaSe. The corresponding stability triangle is shown in Fig. 2. Since La is expected to be in nominal oxidation state +3 in La_2_SeO_2_, we consider Na_L*a*_ as a possible acceptor impurity. (In principle, one could consider K impurities as well. However, the atomic radius of K is larger than the one of La^42^, and so K_L*a*_ will tend to have higher formation energies). We also consider oxygen and selenium vacancies (*V*_O_ and *V*_S*e*_) as possible compensating donor defects. In Fig. 3 we show the formation energies of the three defects, as a function of *E*_*F*_, in La-rich [Fig. 3(a)], with chemical potentials corresponding to point 2 in Fig. 2, and Se-rich conditions [Fig. 3(b)], with chemical potentials corresponding to point 1 in Fig. 2. Only the formation energy of the lowest charge state is shown at any given *E*_*F*_. Figure 3(a) shows that Na_L*a*_ is a shallow acceptor in charge state 2–, but that it will be compensated by *V*_S*e*_, which acts as a deep donor. On the other hand, Fig. 3(b) shows that in Se-rich conditions (and moderately O-rich conditions), 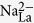 will tend to form spontaneously and that it will not be compensated by either *V*_O_ or *V*_S*e*_. Note that taking the chemical potentials corresponding to point 3 in Fig. 2, will just revert the order of the formation energy curves for *V*_O_ and *V*_S*e*_. This shows that Na-doped La_2_SeO_2_ will indeed behave as a stable *p*-type TCO in strong to moderate anion-rich conditions.

## Discussion

The merit of our list of oxides in Table 1, which present direct band gaps ranging from 3.12 eV to 4.09 eV, and hole effective masses ranging from 0.69 *m*_*e*_ to 0.92 *m*_*e*_, is readily recognised by comparing these with the properties of current *p*-type TCOs. Among the *p*-type oxides reported by Wager and co-authors in ref. 7, Sr-doped LaCuOS, with a direct band gap estimated to be 3.1 eV, presents one of the highest optical transmissions^14^. Note that its band gap value is in the low end of the values in our list of oxides. The *p*-type conductivity of LaCuOS, on the other hand, suffers from a low mobility. Mobility is improved by partially replacing S with Se, raising it from below 1 cm^2^V^−1^s^−1^ up to ~8 cm^2^V^−1^s^−1^ 15, one of the best mobilities in the list of Wager and co-authors^7^. But transparency degrades in the process, as the band gap decreases with Se content down to a vale of 2.8 eV for LaCuOSe^15^. Interestingly, the hole effective mass in LaCuOSe was experimentally estimated to be of ~1.6 *m*_*e*_^29^, which is appreciably higher than the corresponding values in our Table 1. We conclude from the above that one can indeed expect that a suitable synthesis or growth procedure applied to the oxides in Table 1 will result in better *p*-type TCOs, regarding both transparency and hole conductivity.

The *p*-dopability of the oxides in Table 1 can be readily understood in the light of previous work. Indeed, the oxyselenides can be viewed as further examples of the idea of Hosono and co-workers of exploiting the stronger delocalisation of the *p*-orbitals of chalcogens to raise the VBM energy and facilitate *p*-doping^4^. Of course, this is also the reason for their low hole mass.

In Table 1 we indicate the value of the second band gap, which in a *p*-type TCO is defined as the energy difference between VBM and the next eigenvalue below it. This might be of interest in the case of heavily doped TCOs, because at sufficiently high carrier concentration the absorption of photons by the latter will start to be favored and will tend to limit the transparency^43^. However, in active electronic TCO applications (as opposed to passive electronic TCO applications), rather low carrier concentrations are sought^7^, and in that case a low second gap is not an issue.

We mentioned above that in ref. 16 a high-throughput search of low hole mass, wide band gap oxides is performed to identify *p*-type TCO candidates. There is no overlap between the good candidates in ref. 16 and our final list basically because of the different population of oxides studied and because the search criteria are different. Indeed, in ref. 16 no rare-earth containing oxides were considered, and no quaternaries. This excludes the oxides in our Table 1 from their list. On the other hand, of the candidates presented in ref. 16, ZrOS and Na_2_Sn_2_O_3_ are not in our list in Table 1 because of their hole effective masses are higher than 1 *m*_*e*_. PbTiO_3_ and B_6_O do not make it into our list because they do not fall in our class of easily *p*-type dopable oxides. This is in line with a recent first-principles report, which indicates that oxygen vacancies in PbTiO_3_ will tend to act as hole-compensating defects^44^. With respect to B_6_O, which falls in the third class of oxides according to our classification, a recent study on its possible ambipolarity has shown that it indeed will not be easy to dope *p*-type^45^. The only possible *p*-type dopant identified for B_6_O with low enough formation energy near the VBM is a (CH)_O_ complex but it requires at the same time that substitutional carbon in boron position (C_B_) defects are avoided. These results confirm our conclusions regarding their dopability. Finally, K_2_Sn_2_O_3_ and K_2_Pb_2_O_3_ are excluded because their HSE06 direct band gaps are lower than 3.1 eV.

To summarise, in this work we perform a search of new *p*-type TCOs following a high-throughput based on first-principles methods. We first screen all the binary, ternary and quaternary oxides in the AFLOWLIB database to identify those compounds that are reported to have a band gap larger than 2.5 eV, and a hole mass lower than 1 *m*_*e*_. We calculate the electronic structure of the thus identified compounds using state-of-the-art methods in order to determine their *p*-type dopability via the position of their BPE with respect to the band edges. We further require the oxides to have a direct gap larger than 3.1 eV aiming at ensuring their transparency in all the visible energy range. The list of *p*-type dopable, low hole effective mass, transparent oxides that we obtain consists of La_2_SeO_2_, Pr_2_SeO_2_, Nd_2_SeO_2_ and Gd_2_SeO_2_. Furthermore, we explicitly show that in suitable growth conditions Na impurities will behave as shallow acceptors in La_2_SeO_2_, when substituting La, and that anion vacancies will not compensate them. Because of their characteristics, these oxides have the potential to outperform the currently used *p*-type TCO materials and to lead to a breakthrough in transparent electronics applications. Indeed, to our knowledge none of the stable *p*-type TCOs reported so far in the literature has simultaneously a hole effective mass <1 *m*_*e*_ and a band gap >3.1 eV. We hope that experimentalists will be intrigued by our results and will be encouraged to try to confirm our findings.

## Methods

### First-principles calculations

All structural and electronic properties calculations in this work are performed within density functional theory (DFT)^46^,^47^, using the plane-wave basis sets and the projector augmented-wave method^48^, as implemented in the Vienna Ab-initio Simulation Package (VASP)^49^,^50^,^51^,^52^. We use the Heyd, Scuseria, Ernzerhof (HSE06) hybrid functional for the exchange-correlation potential^31^,^32^ (with the standard 25% of exact echange) to calculate the lattice parameters and to relax the atomic positions, as well as to determine the electronic structure and to determine formation energies.

As discussed by Jain *et al.*^53^, in a high-throughput approach, it is impractical to perform rigourous energy cutoff and **k**-point convergence studies during the screening procedure. Thus, we use an energy cutoff of 400 eV for the plane-wave basis set, which is sufficiently high to ensure convergence. To sample the Brillouin zone, we use a Monkhorst-Pack grid^54^, making sure that the Γ point is included in the mesh. For the number of **k**-points in the mesh, we follow ref. 53, sampling the first Brillouin Zone using a grid of at least 500/*n* **k**-points, where *n* is the number of atoms in the unit cell. The convergence tests are performed for the candidates selected at the end of the screening procedure, focusing on the BPE and formation energy. Finally, we note that atomic relaxations are made until residual forces on the atoms are less than 0.01 eV/Å and total energies are converged to within 1 meV.

### Branch point energy

As indicated above, the BPE is calculated as a weighted average of the midgap energies over the Brillouin zone^36^,^37^,


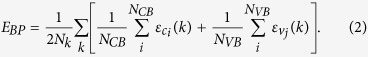


Here, *N*_*k*_ is the number of points in the **k**-point mesh, *N*_*CB*_ and *N*_*VB*_ are the number of conduction and valence bands considered for the averaging, with *ε*_*c*_ and *ε*_*v*_ their corresponding energies. The **k**-point grid is sufficient to have a *E*_*BP*_ converged with respect to the number of **k**-points. The number of valence and conduction bands used is determined by scaling them according to the number of valence electrons in the primitive cell (excluding d electrons), as in the work of Schleife *et al.*^36^. Note that in the latter work it is recognised that the dependence of *E*_*BP*_ on the number of bands used results in an uncertainty of ~0.2 eV on its value. However, given that we consider here wide band oxides (*E*_*g*_ ≤ 2.5 eV), and that we impose a strong criterion, namely that the *E*_*BP*_ should not lie above the VBM by more than 1/4 of *E*_*g*_ , the uncertainty mentioned will not change our conclusions regarding the easy dopability of our selected compounds.

### Defect formation energy and stability triangle

To model the defect system we use a 90-atom 3 × 3 × 2 supercell, and sample the Brillouin zone using a 2 × 2 × 2, Γ-centered, Monkhorst-Pack grid. We denote the chemical potential of the constituent elements *μ*_L*a*_, *μ*_S*e*_, and *μ*_O_. The chemical potentials are related to the enthalpy of formation of the oxide through Δ*H*_*f*_ (La_2_SeO_2_) = 2Δ*μ*_L*a*_ + Δ*μ*_S*e*_ + 2Δ*μ*_O_. As indicated previously, 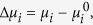 with 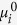 the chemical potential in the standard phase of the element, i.e., metallic La and Se, and molecular O (note that a spin-polarised calculation must be performed in the latter case). In a first instance, the range of possible chemical potential values are determined by the value of the enthalpy of formation and the limits imposed by the precipitation of the constituent elements, i.e., Δ*μ*_L*a*_ = 0 (La-rich), Δ*μ*_S*e*_ = 0 (Se-rich), and Δ*μ*_O_ = 0 (O-rich). This results in the triangular area in the (Δ*μ*_S*e*_, Δ*μ*_O_) plane plotted in Fig. 2, which is known as a stability triangle in the literature^55^. As indicated in Fig. 2, the relevant range of chemical potentials is further limited by phase segregation, i.e., the enthalpy of formation of binary oxides, Δ*H*_*f*_ (L*a*_2_O_3_) = 2Δ*μ*_L*a*_ + 3Δ*μ*_O_ and Δ*H*_*f*_ (L*aSe*) = Δ*μ*_L*a*_ + Δ*μ*_S*e*_. This defines the area within the stability triangle, in which L*a*_2_S*e*O_2_ is stable, and the exact chemical potential values of, e.g., points 1 and 2. These are feeded to Eq. (1) to calculate the formation energies plotted in Fig. 3 in the main article. Note that in Eq. (1) the VBM of the undoped oxide is used as reference (*E*_*v*_) for the electronic chemical potential, and the alignment term (Δ*V*) is calculated following the procedure introduced in ref. 56 (see also ref. 57).

## Additional Information

**How to cite this article**: Sarmadian, N. *et al.* Easily doped *p*-type, low hole effective mass, transparent oxides. *Sci. Rep.* **6**, 20446; doi: 10.1038/srep20446 (2016).

## Supplementary Material

Supplementary Information

## Figures and Tables

**Table 1:** Properties of the easily *p*-type dopable, low hole effective mass, transparent oxides identified via the selection procedure in this work.

oxide	*E*_*g*_	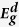	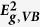 1	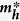	Δ*Hf*^2^
La_2_SeO_2_	3.49	4.02	1.55	0.92	−15.62
Pr_2_SeO_2_	3.26	4.09	1.99	0.69	−15.09
Nd_2_SeO_2_	2.76	3.12	1.76	0.79	−14.72
Gd_2_SeO_2_	3.07	3.95	2.28	0.76	−32.67

The fundamental band gap, *E*_*g*_, first direct band gap, 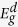, second gap direct gap in the valence band, 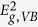, and standard enthalpy of formation, Δ*H*_*f*_, are our HSE06 calculated values. The average hole effective mass 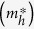 is the AFLOWLIB value. (Energies are in eV, and effective masses are in units of *m*_*e*_).

^1^For the compounds that have a non-zero total magnetic moment 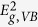 is the energy difference between the two highest occupied bands with the same spin component.

^2^Enthalpy of formation energy per formula unit with respect to the constituent elements in their standard phases.
