# Supplementary material for: Easily doped p-type, low hole effective mass, transparent oxides
Source: Sci Rep. 2016 Feb 8;6:20446. doi: 10.1038/srep20446 (PMC4745066; doi:10.1038/srep20446)
Supplement: Supplementary Information [file srep20446-s1.pdf]

Easily p-type dopable, low hole effective mass, transparent oxides Supplementary Information

Nasrin Sarmadian[1,*], Rolando Saniz[1], Bart Partoens[1], and Dirk Lamoen[2]

[1]CMT, Departement Fysica, Universiteit Antwerpen, Groenenborgerlaan 171, B-2020 Antwerpen, Belgium.
[2]EMAT, Departement Fysica, Universiteit Antwerpen, Groenenborgerlaan 171, B-2020 Antwerpen, Belgium.
[*]nasrin.sarmadian@uantwerpen.be

Supplementary Tables

Table S1. Lattice parameters (a, b, c) (Å), fundamental band gap E_g (eV), and electron and hole effective mass, m_e^*, and m_h^*, respectively (in units of the free electron mass m_e). The lattice parameters in the first row are from the ICSD database. The band gap and effective mass values in the first row are from the AFLOWLIB database. Second row of data presents our HSE06 calculated values.

oxide	structure††	a	b	c	E_g	m_e^*	m_h^*
			**part I**				
$LiNbO_2$	HEX	2.90	2.90	10.46	3.16	2.39	0.76
		2.90	2.90	10.32	2.39		
$NaNbO_2$		2.95	2.95	11.58	2.84	0.90	0.70
		2.95	2.95	11.46	2.32		
Pr_2SO_2†	HEX	3.93	3.93	6.75	4.03	1.11	0.84
		3.94	3.94	6.79	2.61		
Pr_2SeO_2†		4.01	4.01	7.05	3.29	0.88	0.69
		3.97	3.97	7.00	3.26		
Ba_2InFO_3	TET	4.16	4.16	13.94	3.67	0.34	0.78
		4.14	4.14	13.87	3.73		
Ba_2InClO_3		4.22	4.22	15.03	3.50	0.33	0.84
		4.21	4.21	14.95	3.50		
Ba_2InBrO_3		4.24	4.24	15.48	3.38	0.31	0.86
		4.24	4.24	15.54	3.33		
$K_2Sn_2O_3$	BCC	7.28	7.28	7.28	2.67	0.84	0.27
		7.14	7.14	7.14	2.15		
$K_2Pb_2O_3$		7.29	7.29	7.29	3.20	1.24	0.37
		7.22	7.22	7.22	2.66		
$Sb_4Cl_2O_5$	MCL	5.11	6.24	13.54	4.47	3.77	0.69
		5.16	6.40	13.38	3.43		
$Sb_4Br_2O_5$		5.13	6.59	13.43	4.11	1.73	0.70
		5.16	6.46	13.39	3.47		
			**Part II**				
La_2O_3	HEX	6.12	6.12	3.93	3.04	0.56	0.84
		6.27	6.27	3.26	2.68		
B_6O	RHL	5.15	5.15	5.15	3.42	0.37	0.67
		5.12	5.12	5.12	2.85		
$SrNiO_2$†	ORCC	3.58	3.92	8.38	2.55	8.19	0.71
		3.53	3.87	8.35	1.64		
$AgNbO_3$	ORCC	6.63	6.63	6.23	3.39	4.91	0.86
		6.69	6.69	6.12	1.94		
$Rb_2Sn_2O_3$	RHL	6.14	6.14	6.14	2.56	0.44	0.28
		6.05	6.05	6.05	1.96		
BTl_3O_3	HEX	9.27	9.27	3.78	2.76	0.44	0.96
		9.21	9.21	3.66	1.96		
Ca_4As_2O	BCT	4.54	4.54	8.36	2.54	0.29	0.66
		4.53	4.53	8.33	1.97		
$MnWO_4$†	MCL	4.81	5.04	5.83	3.90	28.91	0.68
		4.78	4.96	5.70	2.10		

Continued

oxide	structure††	a	b	c	E_g	m_e^*	m_h^*
$CaNb_2O_4$	ORC	5.05	5.84	11.90	2.70	0.99	0.71
		5.04	5.82	11.83	2.12		
VAg_3O_4	MCLC	5.09	5.83	9.58	2.80	2.14	0.98
		4.96	5.61	9.53	2.14		
Tl_6TeO_6	RHL	6.35	6.35	6.35	2.69	0.40	0.90
		6.59	6.59	6.59	2.33		
$TiPbO_3$	CUB	3.88	4.00	4.21	4.08	38.34	0.79
		3.91	3.91	3.91	2.65		
Na_3AgO_2	ORCI	5.46	5.93	6.79	2.64	0.56	0.92
		5.40	5.85	6.70	2.71		
BiIO	TET	3.98	3.98	9.13	2.94	0.23	0.50
		4.01	4.01	9.47	2.72		
Nd_2SeO_2†	HEX	3.97	3.97	6.98	3.52	0.94	0.79
		3.95	3.96	6.97	2.76		
$Bi_{12}PbO_{20}$	BCC	8.91	8.91	8.91	2.97	1.68	0.89
		8.76	8.76	8.76	3.04		
Gd_2OSe_2†	ORC	3.87	3.87	6.85	2.98	1.19	0.76
		3.87	3.87	6.86	3.07		
$Tl_4V_2O_7$	HEX	5.94	5.94	7.73	4.41	1.93	0.75
		5.88	5.88	7.59	3.10		
Hg_2SeO_3	ORCI	7.83	8.81	8.81	4.06	2.53	0.58
		7.71	8.77	8.77	3.10		
$SbTlO_3$	HEX	5.31	5.31	14.25	3.79	0.61	0.86
		5.29	5.29	13.69	3.12		
SI_2O_6	MCLC	4.69	7.94	8.58	1.48	0.87	0.59
		4.63	7.83	8.28	3.18		
Er_2SeO_2	HEX	3.79	3.79	6.74	3.85	0.61	0.81
		3.79	3.79	6.74	3.19		
Ho_2SeO_2	HEX	3.81	3.81	6.77	3.82	0.65	0.80
		3.78	3.78	6.73	3.19		
$PbSb_2O_6$	HEX	5.30	5.30	5.38	3.54	0.39	0.51
		5.34	5.34	5.35	3.26		
La_2SeO_2	HEX	4.07	4.07	7.12	4.06	0.73	0.92
		4.06	4.06	7.12	3.49		
$CaTe_3O_8$	MCLC	5.66	6.90	9.65	3.61	0.54	0.68
		5.34	5.97	9.54	3.54		
$PbAs_2O_6$	HEX	4.87	4.87	5.48	3.79	0.36	0.40
		4.94	4.94	5.47	3.66		
Hg_2SO_4	MCL	4.43	6.28	8.37		0.55	0.80
		4.36	6.28	8.37	3.17		
$HfPbO_3$	ORC	5.84	5.17	11.71	3.58	0.38	0.99
		5.81	8.09	11.63	3.58		
LaHO	TET	5.74	8.07	8.07	3.87	0.55	0.86
		5.66	8.00	8.00	3.82		
$BiAsO_4$	MCL	6.73	6.88	7.16	3.28	3.00	0.76
		6.55	6.69	7.07	4.88		
$Tb_2Ti_2S_2O_5$	BCT	3.79	3.79	11.74	2.73	0.71	0.47
		3.74	3.74	11.66	1.81		
DyZnPO	RHL	10.35	10.35	10.27	2.83	0.49	0.57
		10.27	10.27	10.27	1.95		

Continued

oxide	structure††	a	b	c	E_g	m_e^*	m_h^*
YZnPO	RHL	10.35	10.35	10.35	2.84	0.51	0.60
		10.30	10.30	10.30	2.02		
$NdBi_2ClO_4$†	TET	3.91	3.91	8.99	2.62	0.28	0.96
		3.89	3.89	8.88	2.19		
$KNdPdO_3$†	MCLC	3.94	6.89	7.37	2.66	0.75	0.28
		3.92	6.82	7.24	2.24		
$NaVS_2O_8$†	MCLC	4.85	4.85	7.19	2.69	2.93	0.67
		4.68	4.68	6.79	2.75		
$TbBi_2ClO_4$	TET	3.86	3.86	8.91	2.82	0.27	0.87
		3.84	3.84	8.82	2.82		
$DyBi_2ClO_4$	TET	3.86	3.86	8.89	2.84	0.27	0.86
		3.83	3.83	8.82	2.85		
Hg_2PFO_3	ORCI	8.57	8.79	8.79	2.86	0.45	0.80
		8.47	8.75	8.75	2.86		
$HoBi_2ClO_4$	TET	3.85	3.85	3.89	2.87	0.27	0.86
		3.82	3.82	8.81	2.88		
$ErBi_2ClO_4$	TET	3.84	3.84	8.88	2.88	0.27	0.86
		3.82	3.82	8.81	2.90		
$ZnTiBi_2O_6$	TET	4.99	5.31	5.31	2.52	0.41	0.96
		4.82	5.20	5.20	3.08		
$Ba_3Bi_2TeO_9$	HEX	6.18	6.18	14.86	3.99	0.56	0.84
		6.16	6.16	14.69	3.09		
$MoNiSr_2O_6$†	BCT	5.58	5.58	5.62	3.65	1.53	0.81
		5.47	5.47	5.51	3.10		
$FeTeFO_3$†	MCL	5.05	5.07	12.40	3.86	3.88	0.77
		4.88	4.92	12.41	3.11		
Ba_2AlInO_5	HEX	5.78	5.78	19.63	3.69	0.38	0.85
		5.81	5.81	19.63	3.39		
$NaVF_2O_2$	MCL	3.59	6.40	7.22	3.90	32.30	0.71
		3.46	6.17	6.88	3.63		
$KGdPdO_3$†	MCLC	3.91	6.80	7.32	3.14	0.75	0.24
		3.89	6.75	7.21	2.32		

†† Acronyms: Monoclinic (MCL), Base Centered Monoclinic (MCLC), Base centered Orthorhombic (ORCC), Body Centered Orthorhombic (ORCI), Tetragonal (TET), Body Centered Tetragonal (BCT), Rhombohedral (RHL), Hexagonal (HEX), Cubic (CUB), Body Centered Cubic (BCC).
† The HSE06 calculated magnetic moment of the compounds that have a non-zero total magnetic moment are available in Table S3.
The results of the compounds that are found metallic with HSE06 (Sn_2F_5O, and $MnMoO_4$) are not included in the table.

Table S2. The calculated data for all the *p*-type dopable oxides, including those that have a direct band band gap smaller than 3.1 eV. For each compound the HSE06 calculated band gaps (fundamental band gap E_g, first direct band gap E_g^d, second gap in the valence band $E^2_{g,VB}$) are compared with the other available results (second row for each compound). The PBE calculated hole effective mass along symmetry directions (m_h^*), average hole effective mass (m_h^{*avg}), and enthalpy of formation ΔH_f are also presented. (All energies are in eV, and all effective masses are in units of the free electron mass m_e)

oxide	E_g	E_g^d	$E^2_{g,VB}$	$m_h^{*\dagger}$	$m_h^{*avg\dagger\dagger}$	$\Delta H_f^{\ddagger}$
$K_2Pb_2O_3$	2.66	2.66	1.55	0.30	0.30	-8.94
Nd_2SeO_2	2.76	3.12	1.76	$0.98^{[100]}$, $0.18^{[001]}$	0.71	-14.72
Gd_2SeO_2	3.07	3.95	2.28	$0.49^{[100]}$, $1.18^{[001]}$	0.72	-32.67
$Tl_4V_2O_7$	3.10	3.13	1.49	$0.53^{[100]}$,$0.97^{[001]}$	0.65	-21.78
$FeTeFO_3$	3.11	3.12	0.41	$0.27^{[100]}$, $1.50^{[010]}$, $0.23^{[001]}$	0.67	-8.87
Pr_2SeO_2	3.26	4.09	1.99	$1.03^{[100]}$, $0.22^{[001]}$	0.76	-15.09
La_2SeO_2	3.49	4.02	1.55	$0.28^{[100]}$, $2.63^{[001]}$	0.59	-15.62
$Tb_2Ti_2S_2O_5$	1.80	1.81	0.52	$0.81^{[001]}$, $0.86^{[100]}$	0.83	-32.53
BTl_3O_3	1.96	2.13	1.25	$0.97^{[100]}$, $0.63^{[001]}$	0.84	-9.22
Ca_4As_2O	1.97	2.42	0.83	$0.51^{[100]}$, $0.97^{[001]}$	0.63	-12.04
DyZnPO	1.95	2.41	0.71	$0.22^{[100]}$, $4.94^{[001]\S}$	0.62	-11.86
$Rb_2Sn_2O_3$	1.96	1.96	1.87	$0.17^{[100]}$, $0.31^{[001]\S}$	0.21	-10.38
YZnPO	2.02	2.07	0.72	$0.23^{[100]}$, $5.40^{[001]\S}$	0.66	-7.62
$MnWO_4$	2.10	2.11	0.37	⋆		-11.99
$CaNb_2O_4$	2.12	2.34	0.31	$0.59^{[100]}$, $0.63^{[010]}$, $0.87^{[001]}$	0.69	-18.49
$K_2Sn_2O_3$	2.15	2.15	2.08	0.21	0.21	-10.39
$NaNbO_2$	2.32	2.40	0.49	$0.45^{[100]}$, $1.16^{[001]}$	0.62	-8.78
$LiNbO_2$	2.39	2.39	0.46	$0.41^{[100]}$, $1.79^{[001]}$	0.67	-9.51

† For materials with a cubic structure, the effective mass is calculated along [100].
†† Average hole effective mass calculated using $m^{*avg} = (m_1 m_2 m_3)^{1/3}$ where m_1, m_2, and m_3 is the hole effective mass along [100], [010], and [001], respectively.
§ For materials with a rhombohedral structure, the effective mass is calculated for the corresponding hexagonal conventional cell.
‡ ΔH_f is the formation energy per formula unit with respect to the metal phase of the elements and molecular oxygen.
⋆ For this compound that is found metallic with PBE, the effective mass is not calculated.

Table S3. HSE06 calculated magnetic moment per formula unit ($\mu/f.u.$) of the oxides that have a non-zero total magnetic moment. The HSE06 values are also compared with the available corresponding values from the AFLOWLIB database. The values are in units of the Bohr magneton μ_B.

oxide	$\mu/f.u.$ (HSE06)	$\mu/f.u.$ (AFLOWLIB)
$SrNiO_2$	4.00	4
Nd_2SeO_2	6.00	6
Pr_2SeO_2	4.00	4
Gd_2SeO_2	14.00	14
Pr_2SO_2	4.00	4
$FeTeFO_3$	5.00	5
$KGdPdO_3$	7.00	7
Bi_2NdClO_4	3.00	3
$KNdPdO_3$	3.00	3
$MnWO_4$	5.00	5
$MoNiSr_2O_6$	2.00	2
$NaVS_2O_8$	2.00	2

Supplementary Figures

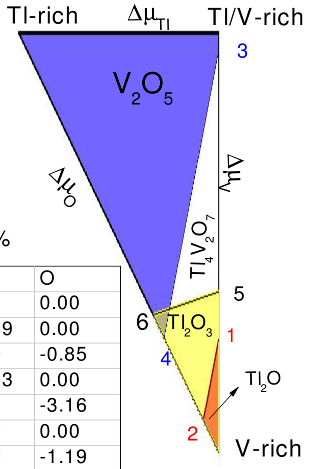

Tl4V2O7	Tl	V	O
Tl-poor	-5.44	0.00	0.00
V-poor	0.00	-10.89	0.00
point1	0.00	-7.89	-0.85
point2	-0.43	-10.03	0.00
point3	-0.09	0.00	-3.16
point4	-1.49	-7.90	0.00
point5	0.00	-6.72	-1.19
point6	-1.78	-7.32	0.00

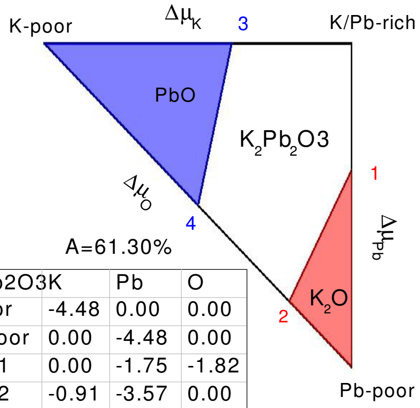

K2Pb2O3K		Pb	O
k-poor	-4.48	0.00	0.00
Pb-poor	0.00	-4.48	0.00
point1	0.00	-1.75	-1.82
point2	-0.91	-3.57	0.00
point3	-1.75	0.00	-2.25
point4	-2.23	-2.25	0.00

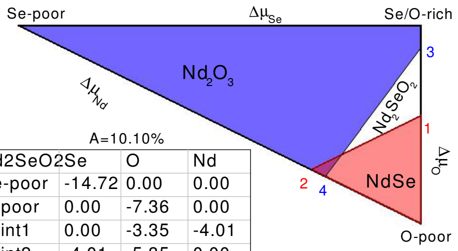

Nd2SeO2Se		O	Nd
Se-poor	-14.72	0.00	0.00
O-poor	0.00	-7.36	0.00
point1	0.00	-3.35	-4.01
point2	-4.01	-5.35	0.00
point3	0.00	-0.81	-8.41
point4	-3.50	-6.88	0.00

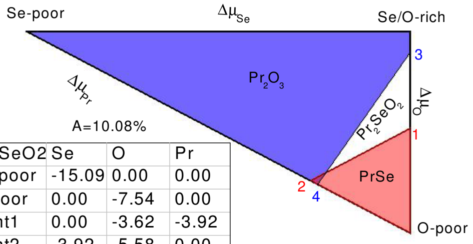

Pr2SeO2	Se	O	Pr
Se-poor	-15.09	0.00	0.00
O-poor	0.00	-7.54	0.00
point1	0.00	-3.62	-3.92
point2	-3.92	-5.58	0.00
point3	0.00	-0.77	-8.58
point4	-3.65	-5.72	0.00

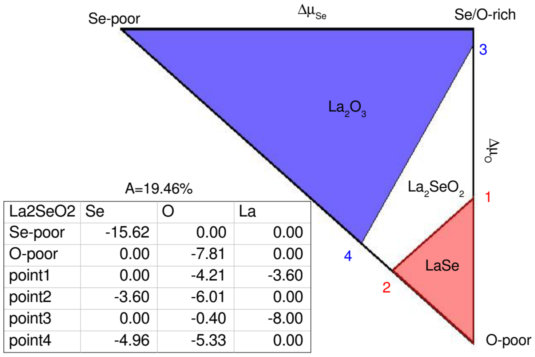

La2SeO2	Se	O	La
Se-poor	-15.62	0.00	0.00
O-poor	0.00	-7.81	0.00
point1	0.00	-4.21	-3.60
point2	-3.60	-6.01	0.00
point3	0.00	-0.40	-8.00
point4	-4.96	-5.33	0.00

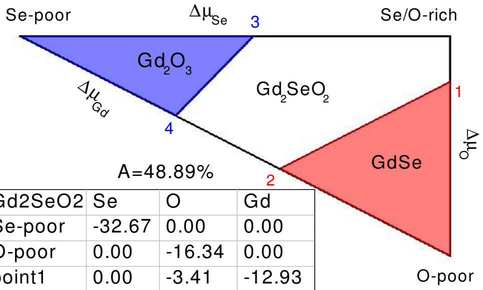

Gd2SeO2	Se	O	Gd
Se-poor	-32.67	0.00	0.00
O-poor	0.00	-16.34	0.00
point1	0.00	-3.41	-12.93
point2	-12.93	-9.87	0.00
point3	-14.87	0.00	-8.90
point4	-20.80	-5.93	0.00

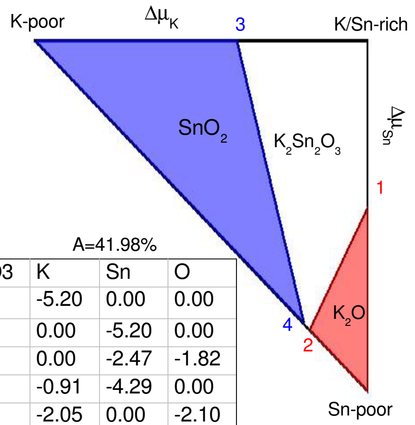

A=41.98%

K2Sn2O3	K	Sn	O
k-poor	-5.20	0.00	0.00
Sn-poor	0.00	-5.20	0.00
point1	0.00	-2.47	-1.82
point2	-0.91	-4.29	0.00
point3	-2.05	0.00	-2.10
point4	-1.00	-4.19	0.00

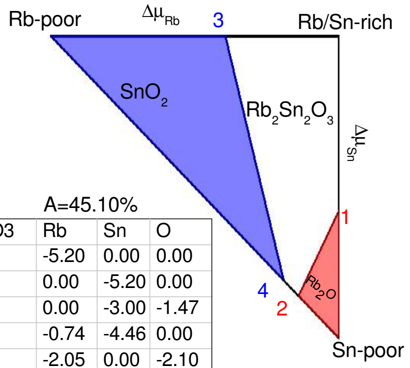

A=45.10%

Rb2Sn2O3	Rb	Sn	O
Rb-poor	-5.20	0.00	0.00
Sn-poor	0.00	-5.20	0.00
point1	0.00	-3.00	-1.47
point2	-0.74	-4.46	0.00
point3	-2.05	0.00	-2.10
point4	-1.00	-4.19	0.00

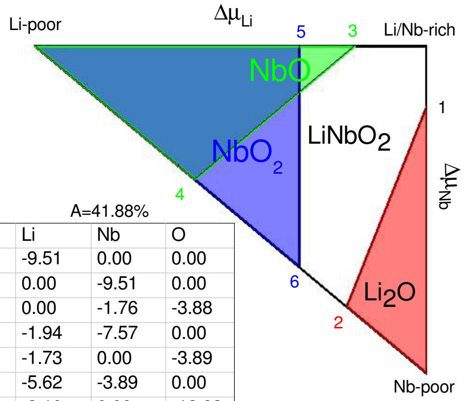

A=41.88%

LiNbO2	Li	Nb	O
Li-poor	-9.51	0.00	0.00
Nb-poor	0.00	-9.51	0.00
point1	0.00	-1.76	-3.88
point2	-1.94	-7.57	0.00
point3	-1.73	0.00	-3.89
point4	-5.62	-3.89	0.00
point5	-3.10	0.00	-12.83
point6	-3.10	-6.41	0.00

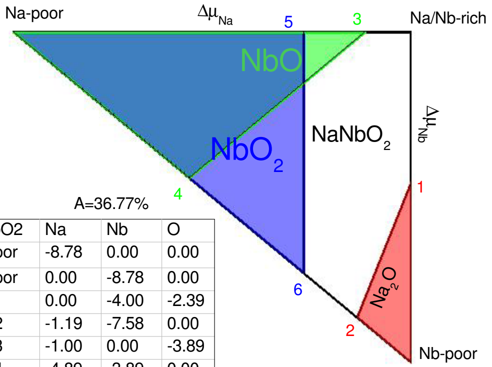

A=36.77%

NaNbO2	Na	Nb	O
Na-poor	-8.78	0.00	0.00
Nb-poor	0.00	-8.78	0.00
point1	0.00	-4.00	-2.39
point2	-1.19	-7.58	0.00
point3	-1.00	0.00	-3.89
point4	-4.89	-3.89	0.00
point5	-2.36	0.00	-12.8
point6	-2.36	-6.41	0.00

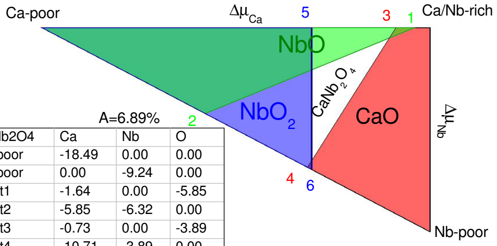

A=6.89%

CaNb2O4	Ca	Nb	O
Ca-poor	-18.49	0.00	0.00
Nb-poor	0.00	-9.24	0.00
point1	-1.64	0.00	-5.85
point2	-5.85	-6.32	0.00
point3	-0.73	0.00	-3.89
point4	-10.71	-3.89	0.00
point5	-5.66	0.00	-3.21
point6	-5.66	-6.41	0.00

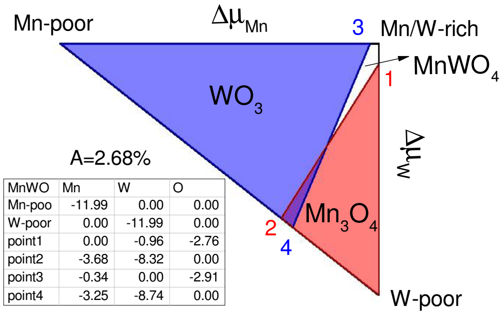

A=2.68%

MnWO	Mn	W	O
Mn-poo	-11.99	0.00	0.00
W-poor	0.00	-11.99	0.00
point1	0.00	-0.96	-2.76
point2	-3.68	-8.32	0.00
point3	-0.34	0.00	-2.91
point4	-3.25	-8.74	0.00

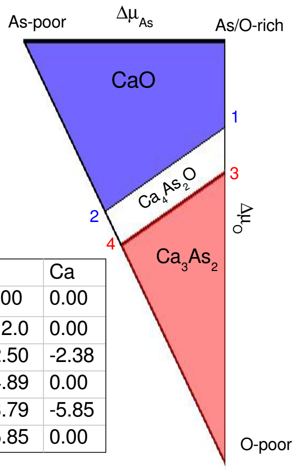

A=11.82%

Ca4As2O	As	O	Ca
As-poor	-6.02	0.00	0.00
O-poor	0.00	-12.0	0.00
point1	0.00	-2.50	-2.38
point2	-3.57	-4.89	0.00
point3	0.00	-3.79	-5.85
point4	-3.09	-5.85	0.00

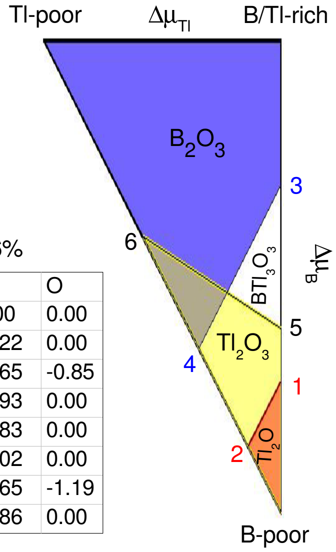

A=23.26%

BTl3O3	Tl	B	O
Tl-poor	-3.07	0.00	0.00
B-poor	0.00	-9.22	0.00
point1	0.00	-6.65	-0.85
point2	-0.43	-7.93	0.00
point3	0.00	-2.83	0.00
point4	-1.06	-6.02	0.00
point5	0.00	-5.65	-1.19
point6	-1.48	-3.86	0.00

Figure S1. PBE calculated stability triangle of the *p*-type dopable ternary oxides. Each plot indicates the range of the chemical potentials for which the ternary oxide is stable against the formation of the competing phases[a,b,c]. For each compound, the chemical potential values (in eV) at the points delimiting the different stability areas are shown in the table next to the triangle.

[a] All the binaries that may contribute in synthesis of a ternary are considered as competing phases.

[b] We ensure that the most common phase of each binary is considered as a competing phase (e.g. Tl_2O_3 compared to Tl_2O for BTl_3O_3) is considered as a competing phase.

[c] For clarity, we only show the binary oxide for which the stability area is not completely covered by that of other considered binary oxides.(e.g. the stability area of Tl_2O is completely covered by that of Tl_2O_3 in the case of BTl_3O_3.)
